# The insulo-opercular cortex encodes food-specific content under controlled and naturalistic conditions

**DOI:** 10.1038/s41467-021-23885-4

**Published:** 2021-06-14

**Authors:** Yuhao Huang, Bina W. Kakusa, Austin Feng, Sandra Gattas, Rajat S. Shivacharan, Eric B. Lee, Jonathon J. Parker, Fiene M. Kuijper, Daniel A. N. Barbosa, Corey J. Keller, Cara Bohon, Abanoub Mikhail, Casey H. Halpern

**Affiliations:** 1 Department of Neurosurgery, Stanford University, Stanford, CA USA; 2 Departments of Psychiatry and Behavioral Sciences, Stanford University, Stanford, CA USA; 3 Veterans Affairs Palo Alto Healthcare System, and the Sierra Pacific Mental Illness, Research, Education, and Clinical Center (MIRECC), Palo Alto, CA USA

**Keywords:** Neuroscience, Cognitive neuroscience, Feeding behaviour, Gustatory system, Neural circuits

## Abstract

The insulo-opercular network functions critically not only in encoding taste, but also in guiding behavior based on anticipated food availability. However, there remains no direct measurement of insulo-opercular activity when humans anticipate taste. Here, we collect direct, intracranial recordings during a food task that elicits anticipatory and consummatory taste responses, and during ad libitum consumption of meals. While cue-specific high-frequency broadband (70–170 Hz) activity predominant in the left posterior insula is selective for taste-neutral cues, sparse cue-specific regions in the anterior insula are selective for palatable cues. Latency analysis reveals this insular activity is preceded by non-discriminatory activity in the frontal operculum. During ad libitum meal consumption, time-locked high-frequency broadband activity at the time of food intake discriminates food types and is associated with cue-specific activity during the task. These findings reveal spatiotemporally-specific activity in the human insulo-opercular cortex that underlies anticipatory evaluation of food across both controlled and naturalistic settings.

## Abstract (web summary)

Animal studies have shown that insulo-opercular network function is critical in gustation and in behaviour based on anticipated food availability. The authors describe activities within the human insulo-opercular cortex which underlie anticipatory food evaluation in both controlled and naturalistic settings.

## Introduction

To make perceptual inferences and guide behavior, the human brain relies on the discriminatory processing of sensory information across domains. Environmental cues which indicate food availability thus drive food-seeking and eating behavior^1^. Moreover, the presentation of highly palatable foods such as high-fat and high-sugar items can provoke food intake even in periods of relative energy abundance^2^. Not surprisingly, dysregulation of this process can result in pathologic conditions such as eating disorders and obesity^3,4^.

A number of rodent studies have shed light on the real-time spatiotemporal dynamics of taste and food processing and its link to eating behavior. Information from various visceral and gustatory inputs has been reported to converge on the insular cortex, which then generates a homeostatic response to food cues^5^. These inputs include the lateral hypothalamus, amygdala, and nucleus accumbens^6–9^. Temporally-specific inactivation of the insular cortex using optogenetics abolished cue-provoked food-seeking activity^10^. In addition, human neuroimaging studies have suggested the involvement of the insular cortex and the overlying frontal operculum in taste evaluation and in representation of cues associated with food availability, a process that is dysregulated in the obese state^11–13^.

Although in vivo rodent studies reveal the critical involvement of this cortical region in a process required for survival, they do not readily allow examination of neural processing of complex symbolic and/or language cues encountered in society. Current neuroimaging techniques in humans have limited temporal resolution, thus limiting precise characterization of insulo-opercular dynamics during eating behavior.

The aim of this study was to address this gap in knowledge by measuring the activity of human frontal opercular and insular cortices underlying food processing. Specifically, we were interested in a better understanding of the real-time physiologic responses during food anticipation. We hypothesized both food-specific and topology-specific anticipatory responses within the insulo-opercular cortex. To test our hypothesis, we leveraged invasive brain recordings from depth electrodes in epilepsy patients as they performed a measure of anticipatory and consummatory food rewards. To assess whether findings related to this task could be generalized to a naturalistic setting, where phases of food anticipation and consummation are not distinctly separated, we also utilized recordings during epochs of ad libitum consumption captured by video recordings of regular meals. We hypothesized that regions of the insulo-opercular cortex that exhibit food-cue-specific activity would also be involved in an expectant evaluation of food during regular meal consumption.

## Results

Eleven subjects (2 females) participated in the task paradigm (Table 1). All subjects were right-handed. All subjects reported they preferred the palatable solution over the taste-neutral solution, with an average rating of 6.1.

**Table 1:** Participant characteristics.

ID	Age	Seizure focus	BMI	Palatable liquid rating	Preferred palatable over neutral?	Number of contacts		
						Frontal operculum	Ant. Insula	Pos. Insula
S1	59	Bitemporal	26	5/10	Yes	2(L) 2(R)	6(L) 5(R)	1(L) 2(R)
S2	34	L. Lateral Temporal	32	3/10	Yes	0	9(L)	0
S3	24	R. Mesial Temporal	51	3/10	Yes	0	9(R)	6(R)
S4	49	L. Frontal and L. Mes. Temporal	31	8/10	Yes	7(L)	1(L)	7(L)
S5	51	L. Mesial Temporal	27	10/10	Yes	2(L)	9(L) 5(R)	4(L)
S6	20	R. Parietal/Occipital	19	5/10	Yes	0	9(R)	0
S7	27	R. Parietal	24	8/10	Yes	1(L)	7(L)	3(L)
S8	36	Bitemporal	23	7/10	Yes	2(L)	7(L)	6(L)
S9	46	R Mesial Frontal	28	5/10	Yes	4(R)	3(R)	0
S10	29	R Frontal Dysplasia	41	3/10	Yes	2(L) 1(R)	8(L) 4(R)	6(R)
S11	62	No sz, pain mapping	29	10/10	Yes	3(L)	10(L) 6(R)	6(L) 3(R)

### Dynamics of neural activity during task paradigm

**Fig. 1:**
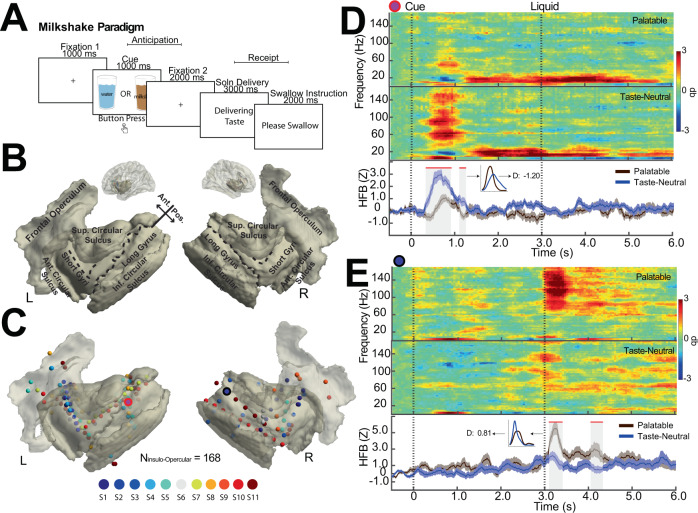
The milkshake paradigm evokes responses in the human frontal opercular and insular cortices. **A** The task paradigm. All subjects performed a food task that involved responding to cues of either palatable (chocolate milkshake) or taste-neutral (water-based solution) liquid and subsequently receiving 3 mL bolus. The anticipatory phase of the task was defined as the onset of the cue immediately prior to solution delivery (0 to 3 s). The receipt phase of the task was defined from solution delivery to immediately prior to swallowing (3 s to 6 s). **B** Freesurfer parcellation of the insular/opercular cortex. The insula was divided into anterior-posterior subregions based on the central sulcus of the insula. The anterior insula includes the short gyri, the anterior (ant.) circular sulcus, and the superior (sup.) circular sulcus; the posterior insula includes the long gyrus and the inferior (inf.) circular sulcus. The frontal operculum is defined as the inferior frontal gyrus. **C** Magnetic resonance image reconstruction of the insular cortex and the frontal operculum overlaid with the translucent cortical surface. All insular/opercular recording contacts are plotted as 3D spheres, color-coded according to subjects, in the Montreal Neurological Institute (MNI) space. **D** Time-frequency spectrogram (decibel, db) and high-frequency broadband (HFB) activity waveform from an insular electrode in a left long gyrus (red circled electrode in **C**) showing differential responses to palatable vs. taste-neutral cue during anticipation. Time points significantly different between palatable and neutral conditions (*p* < 0.05, cluster-based permutation testing, *α* < 0.05) are marked in red along the horizontal axis. The effect size was calculated using time periods that were considered significant as marked by red. The vertical dashed line denotes the onset of the solution cue (*t* = 0 s) and solution delivery (*t* = 3 s). Inset: distribution of mean HFB activity stratified by palatable and neutral trials with the effect size indicated. **E** Identical data representation as shown in **D** for an electrode in the right superior circular sulcus of the insula (black circled electrode in **C**) showing differential responses to palatable vs. taste-neutral solution during receipt. Error bars show ±SEM.

The task consisted of two phases: an anticipatory phase that evaluated neural representation of food expectation and a receipt phase that assessed the neural activity of the sensory and evaluative aspects of food consumption (Fig. 1A). We performed stereoelectroencephalography (SEEG) electrode recording during these two phases with coverage of bilateral insular and frontal opercular cortices across eleven subjects (Fig. 1C). There were 103 electrodes in the left hemisphere and 65 electrodes in the right hemisphere with a total of 168 electrodes covering the insulo-opercular cortex. In the insular cortex, we found electrodes encoding the robust HFB responses to cue or receipt of the solution (Fig. 1D, E). For example, in the left posterior insula, we observed an increase in the HFB activity within 1 s of cue onset, which was greater in response to the taste-neutral cue compared to the palatable cue (Fig. 1D, *p* < 0.05, cluster-based permutation testing, *α* < 0.05, *d* = −1.20). This was also evident in the time-frequency spectrogram. Interestingly, although there was a strong response to the cue at this site, there was no activity upon solution delivery. At a separate site in the anterior insula, instead of an anticipatory response, we observed an increase in the HFB activity at 3 to 3.5 s, corresponding to the time of solution delivery. The palatable solution elicited a greater increase in the HFB activity upon receipt (Fig. 1E, *p* < 0.05, cluster-based permutation testing, *α* < 0.05, *d* = 0.81).

To examine the overall activity of the insulo-opercular cortex during the task, we also computed the time-frequency spectrogram by averaging across all electrodes (*N* = 168, Supplementary Fig. 1A). Again, we observed an increase in broadband power primarily within the first second following either cue presentation (onset at 0 s) or solution receipt (onset at 3 s). There was qualitatively higher broadband power roughly 25–200 Hz in the palatable condition as compared to the taste-neutral condition after cue and receipt. To quantify the difference in neural activity between these two conditions, we calculated effect size in the six traditional band-power frequencies and across all channels in the first second of stimulus onset (Supplementary Fig. 1B). In the anticipatory period, the palatable, compared to taste-neutral, cue-elicited significantly greater average insulo-opercular activity in gamma (*p* = 0.003, one-sample *t*-test, *t* (167) = 3.05) and high-frequency broadband powers (*p* = 0.007, *t*(167) = 2.75). Out of the lower frequency power ranges, only the theta range was associated with higher activity in the palatable condition (*p* = 0.035, t(167) = 2.12). Similarly in the receipt period, delivery of palatable, compared to taste-neutral, liquid to the subjects’ mouth resulted in higher insulo-opercular activity in gamma (*p* = 0.039, two-sample *t*-test, *t*(167) = 2.08) and high-frequency broadband powers (*p* = 0.003, *t*(167) = 3.03). Overall, these results suggest discriminatory neural activity exists in the insulo-opercular region and can be captured by the high-frequency power ranges. Given HFB activity is known to correlate with the blood oxygenation level-dependent (BOLD) signal^14^ and has a known origin from the cortical pial surface^15^, we focused the remaining analyses using HFB activity.

### Topology of insular and frontal opercular responses

Given the overall increase in insulo-opercular activity in the palatable condition (Supplementary Fig. 1), but the variable pattern of response profile in individuals channels (Fig. 1D, E), we sought to determine site-by-site responses driving the group discriminatory activity in the insulo-opercular cortex. We first qualitatively visualized the HFB effect size map on a per-channel basis and used automatic cortical parcellation to determine cortical subregions (Supplementary Fig. 2). In the anticipatory period, we observed higher HFB for the palatable cue (mostly warm colors) as compared to the taste-neutral cue (Supplementary Fig. 2A). Interestingly, we note that there was a localized area of cold colors in the left posterior insular along the dorsal central sulcus, denoting higher HFB activity for the taste-neutral cue. In the receipt period, we observed a heterogeneous distribution of discriminatory HFB profiles, with a predominance of channels showing higher HFB activity for the palatable cue (Supplementary Fig. 2B). Next, we visualized the corresponding HFB activity trace of the labeled electrodes (Supplementary Fig. 2C) to show examples denoting the variation in the pattern of discriminatory responses in the insulo-opercular cortex. These traces were stratified in three columns by whether the electrode was cue-specific, receipt-specific, or non-discriminatory/inactive. We observed that again during the anticipation period, HFB activity generally increased to peak within the first second (Supplementary Fig. 2C, first column). Notably, the left posterior insular responses were largely uniform during anticipation, showing lower HFB activity to the taste-neutral cue as compared to the palatable cue. In contrast, the HFB activity trace in receipt-specific regions was largely heterogeneous, characterized by variable peak latency and waveform morphology (Supplementary Fig. 2C, second column). For reference, we visualized the HFB activity trace of cue-responsive, but not cue-specific sites, and of non-active sites (Supplementary Fig. 2C; third column).

**Fig. 2:**
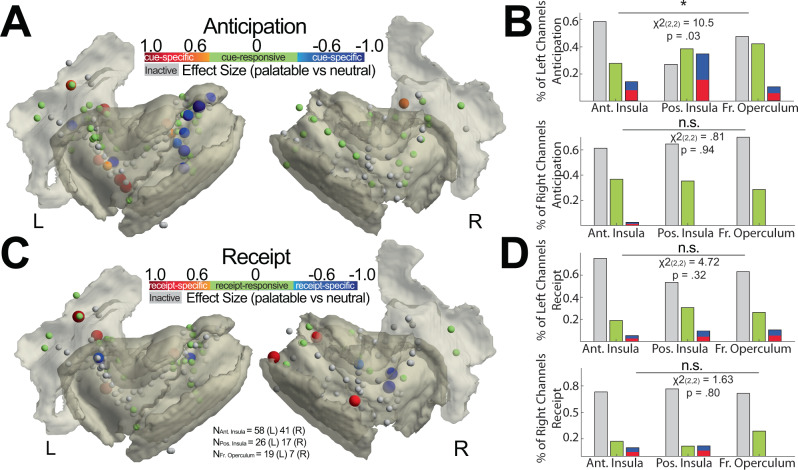
Topographic differences in insular and frontal opercular neural activity when anticipating and receiving task solutions. **A** Site-by-site differences in anticipatory neural activity. Effect size between the anticipatory response to palatable vs. neutral solution is shown per electrode. Shades of red indicate significantly greater response in palatable trials whereas shades of blue indicate significantly greater response in taste-neutral trials. Green indicates a significant increase in activity from pre-cue baseline activity, but no difference between palatable or neutral conditions (cue-responsive but not cue-specific). Gray indicates activity was not different from pre-cue baseline activity (inactive). **B** Proportion of inactive (gray bar), responsive but not specific (green bar), and specific (blue/red bar) electrodes during anticipation stratified by anatomical location and sidedness. Chi-square proportional test was employed. **C**, **D** the Same set of plots as in (**A**, **B**) showing site-by-site differences in receipt neural activity. Chi-square proportional test was employed.

To understand the topology of insulo-opercular activity, we applied a cluster-permutation-based threshold of HFB activity to categorize each electrode as cue/receipt-responsive, cue/receipt-specific, or non-active (Fig. 2). During task anticipation, we found a cluster of cue-specific sites showing greater activity for taste-neutral conditions (cold colors) along the dorsal aspect of the left posterior insula (Fig. 2A left panel). In contrast, the left anterior insula showed several channels with cue-specific activity biased towards palatable conditions (warm colors). The right insular and frontal opercular cortices showed minimal cue-specific activity, with only one channel noted to be cue-specific (Fig. 2A right panel). The left insulo-opercular region was observed to have a significantly different distribution of cue-responsive, cue-specific, or non-active channels, with the highest percentage of cue-specific channels found in the left posterior insula (Fig. 2B top panel; shades of blue or red electrodes; *p* = 0.03, chi-squared test, *χ*2 (2,2) = 10.5). Specifically, the left posterior insula cortex was associated with the highest proportion of taste-neural, cue-specific sites (Supplementary Fig. 3A; *p* = 0.005, *χ*2 (1,1) = 8.03). Amongst cue-specific channels in the anterior and posterior insula, we found the effect size between palatable and taste-neutral conditions was significantly different within these two insular subregions (Supplementary Fig. 3B; *p* = 0.002, two-sample *t*-test, *t*(14) = 3.75). The left posterior insula showed greater activity to the taste-neutral cue, whereas the left anterior insula showed greater activity to the palatable cue. On the right insulo-opercular side, there was no difference in the proportion of distribution in cue-responsive, cue-specific, or non-active sites (Fig. 2B bottom panel; all *p* > 0.05, shades of blue or red electrodes; chi-squared test).

Next, we examined the distribution of responses during task receipt (Fig. 2C). Receipt-responsive and receipt-specific HFB activity was found in a distributed pattern across bilateral insular and frontal opercular cortices. Receipt-responsive, receipt-specific, and non-active channels were distributed in an equal manner across the anterior insula, the posterior insula, and the frontal operculum (Fig. 2D; *p* > 0.05, chi-squared test). In addition, we computed the proportion of task-responsive and task-specific channels in the gamma  band (25–50 Hz) which had also demonstrated higher effect sizes favoring the palatable condition. Compared to HFB activity, a significantly lower number of channels were cue-responsive in the gamma band (Supplementary Fig. 4A; *p* = 0.003, paired *t*-test, *t*(10) = 3.87). Cue-specific channel proportion was also lower for gamma band compared to the HFB, but this was not statistically significant (*p* = 0.08, *t*(10) = 1.95). In addition, the number of receipt-responsive (Supplementary Fig. 4B; *p* = 0.005, *t*(10) = 3.50) channels were significantly lower using gamma band compared to HFB power. However, while receipt-specific channel proportion was also lower for the gamma band, it was not statistically significant (*p* = 0.09; *t*(10) = 1.86). Individual subject topology of the response distribution is available in Supplementary Fig. 5. In addition, the MNI coordinate of every cue-specific and/or receipt-specific electrode has been provided in Supplementary Table 2.

Finally, neural responses to food have been previously reported to depend on individual body mass index (BMI)^11^. We thus investigated if the proportion of responses found in the current study cohort was confounded by individual BMI and the rating of milkshake. Neither the proportion of cue-specific nor cue-responsive channels were associated with subject BMI or the post-task rating of milkshake (Pearson’s Correlation test; all *P* > 0.05; Supplementary Table 1). In addition, no relationships in receipt responses and subject attributes were found (Pearson’s Correlation test; all *P* > 0.05; Supplementary Table 1). Similarly, no correlation between the age of subjects and task responses was found (Pearson’s Correlation test; all *P* > 0.05; Supplementary Table 1).

Taken together, we found evidence of food-specific cue encoding predominantly in the left posterior insular cortex. The left posterior insula cortex tended to favor taste-neutral cues whereas the left anterior insular cortex showed preferential activity for the palatable cue. The encoding of solution receipt was more heterogeneous across the insular and opercular cortices.

### Classification of anticipatory response on a single trial basis

**Fig. 3:**
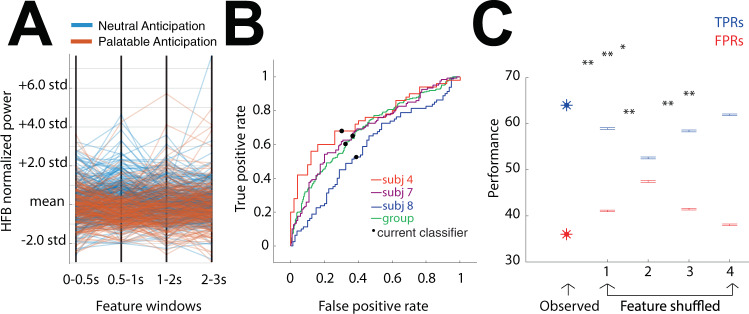
Posterior insular HFB activity is sufficient to classify between anticipation for palatable and taste-neutral solutions on a single trial basis. **A** Parallel coordinates plot displaying normalized HFB power for each observation (*n* = 250 taste-neutral and *n* = 250 palatable trials) as a function of the four features used (4 anticipation time epochs: 0–0.5 and 0.5–1 s of cue presentation, and 1–2 and 2–3 s of post-cue fixation). Note that at the second feature, a cluster of taste-neutral anticipation trials is observed. **B** Inter (green) and intra-individual Receiver Operating Curves (ROCs) for positive class 1 (neutral anticipation). Inter-individual mean TPR (True Positive Rate) and FPR (False Positive Rate) and AUC (Area Under the Curve) were 64%, 36, and 69%, respectively. Intra-individual mean TPR and FPR, and AUC for the three subjects were (4) 69%, 31%, and 74% (7) 64%, 36%, and 69%, and (8) 57%, 43%, and 58% respectively. **C** Statistical testing on classifier performance (TPR in blue, FPR in red). The first set of values represent group classification performance on observed data: 64% mean TPR and 36% mean FPR across the two classes. Same performance measures were computed following permutations (*n* = 100) of values for a given feature across the two classes. Shuffled features 1, 2, 3, and 4 values yielded the following mean TPR and FPR values: (1) 58.84%, 41.06, (2) 52.56, 47.46, (3) 58.43, 41.46, and (4) 61.94, 38.06. Error bars represent S.E.M. across performance measures on shuffled data (*n* = 100). Observed TPR and FPR significantly differed from features 1, 2, and 3 shuffled data (*p* < 0.01, *p* < 0.01, and *p* = 0.01, respectively). Feature 4 shuffled data did not significantly affect classification performance (*p* = 0.08). Performance using feature 2 shuffled data was significantly diminished compared to performance on shuffled data for any other feature (Tw-sample *t*-test, corrected for multiple comparisons, *p* = 0.02 for features 2 vs. 1, 3, and 4, *p*_FDR_ = 0.002). *p < 0.05, **p < 0.01.

Taste-neutral HFB responses were relatively localized to the posterior insula. We, therefore, tested whether posterior insular HFB activity was sufficient to classify between the two anticipatory conditions on a single trial basis (Fig. 3). To do so, a weighted KNN classifier was trained and tested on data from cue-responsive (displaying significantly increased HFB power from baseline) and cue-specific (displaying significantly higher power for taste-neutral compared to palatable anticipation) posterior insula channels. The parallel coordinate plot displays all utilized observations (*n* = 500) stratified by palatable and taste-neutral trials, as a function of the features utilized (Fig. 3A). A 4-D feature vector used which included mean HFB power averaged in four anticipatory time epochs (Fig. 3A; 0–0.5 s, 0.5–1.0 s, 1.0–2.0 s, 2.0–3.0 s). PCA was used for dimension reduction; after training, four components were kept to explain 95% of the variance (explained variance per component, in order, was 61.8%, 16.3%, 13.6%, and 8.2%). Notably, we observed a cluster of taste-neutral observations in the 0.5–1.0 s feature window (Fig. 3A).

Inter-individual (group) classification performance yielded 64% mean TPR (True Positive Rate) and 37% mean FPR (False Positive Rate) across the two classes (Fig. 3B), with an AUC of 69%. An individual subject model was also generated, separately for each of the three subjects. Mean TPR, FPR, and AUC for each of the three subjects were (S4) 69%, 31%, and 74% (S7) 64%, 36%, and 69% and (S8) 57%, 43%, and 58%, respectively (Fig. 3B). Note that for all classifiers, mean TPR across classes and overall accuracy are equal due to a matched observation number in both classes. The classifier depended significantly on three of the four time periods (*p* < 0.01, *p* < 0.01, and *p* = 0.01, respectively), while reliance on the fourth time period did not reach significance (*p* = 0.08). Classification performance was significantly more diminished when permuting the second time period compared to any other time period (Fig. 3C; *p* = *0.002* feature 2 vs. 1*, p* = *0.002* feature 2. vs. 3, *p* = *0.002* feature 2. vs. 4, *P*_FDR_ = 0.002). This suggests that HFB activity during food anticipation is sufficient to classify between palatable and taste-neutral conditions, with the 0.5 to 1.0 s period following cue being most contributory to classification.

### The sequence of insular and frontal opercular activity

**Fig. 4:**
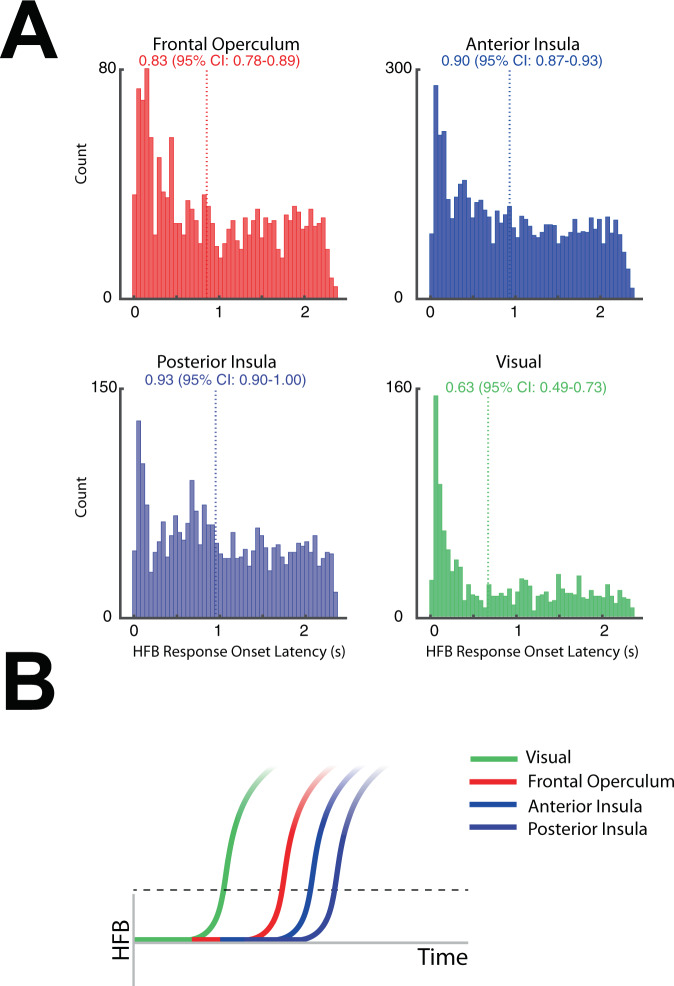
Comparison of HFB response onset by anatomical location during task anticipation. **A** Histogram showing the distribution of response onset latency (ROL) of HFB at the frontal operculum, the anterior insula, the posterior insula, and visual regions. Each observation represents the ROL of HFB at the single-trial level calculated for all trials across all subjects (palatable and taste-neutral conditions). The vertical line denotes the median ROL time. **B** Schematic representing the sequence of HFB activity onset: early visual response to cue with the subsequent delay in activation of the frontal operculum, followed by involvement of anterior insula and subsequently posterior insula.

Next, to better define the latency of HFB activity at each anatomic region we characterized the HFB response onset latency (ROL) during anticipation and receipt. Specifically, using a single trial approach, we determined the HFB ROL at the frontal operculum, the anterior insular cortex, and the posterior insular cortex. During anticipation, we found earlier response onset in the frontal operculum (Fig. 4A, median, 95% CI: 830 ms, 780–890 ms) compared to the posterior insular cortex (930 ms, 900–1000 ms). The anterior insula had a response onset in between (900 ms, 870–930 ms). As a control, we determined the HFB ROL in visual sites which would be expected to respond to the visual cue^16^. As expected, the visual sites showed the earliest onset time ( 630 ms, 490–730 ms). In contrast, during task receipt, the distribution of the HFB ROL across sites was flat with no obvious peak onset time (Frontal Operculum: 4220 ms, 4150–4310 ms; Anterior Insula: 4290 ms, 4250–4320 ms; Posterior Insula: 4200 ms, 4150–4260 ms; Visual 4080 ms, 3990–4170 ms; Supplementary Fig. 6).

### A naturalistic examination of dynamic, time-locked neural activity during eating

As most human studies involving feeding behavior are performed within the confines of a controlled task setting, little is known about how activity during a task may be relevant to neural dynamics during eating in the natural setting. As subjects in the epilepsy monitoring unit were continuously video monitored, this afforded us a unique opportunity to investigate neural activity when subjects consumed meals during their hospital stay. We hypothesized that the left posterior insular cortex, which was found to be consistently cue-specific during task anticipation, may encode similar activity during consumption of a regular meal.

**Fig. 5:**
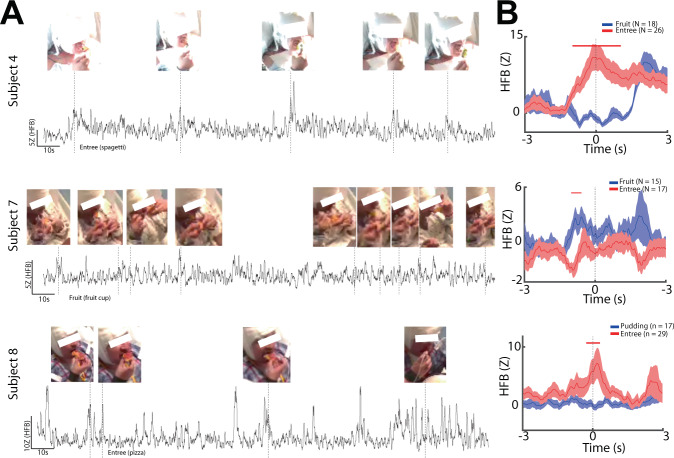
Time-locked HFB activity during natural eating stratified by type of food consumed. **A** Simultaneous HFB activity trace and video capture during meal consumption. For each subject, HFB activity computed from spike-minimized data in an exemplar electrode in the left posterior insula is shown. Dotted vertical lines denote the moment food was about to enter the subject’s mouth, with accompanying images showing the time point that was used for time-locking. The food being consumed is denoted under the activity trace. **B** Mean time-locked HFB waveforms stratified by type of food consumed. Dotted vertical lines denote the immediate time point as the food was about to enter the subject’s mouth. Time points significantly different between entrée vs. non-entrée near the time of food entering the mouth (*p* < 0.05, cluster-based permutation testing, *α* < 0.05) are marked in red along the horizontal axis. Error bars show ±SEM.

We identified three (subjects 4, 7, 8) subjects with at least two or more cue-specific channels in the left posterior insular cortex. In these three subjects, we identified video segments for analysis of ad libitum consumption (Fig. 5). We reasoned a repeated anticipatory moment during eating might be when food is brought from the plate to the mouth, at which time subjects might preemptively perceive the various sensory aspects of the food. As such, we used the time point immediately preceding food entering the mouth as a time-lock (Fig. 5A; vertical dotted lines). In the included meal sessions, subject 4 took 26 bites of an entrée dish (rice with assorted vegetables and meat) and 18 bites of a fruit cup; subject 7 took 17 bites of an entrée dish (pasta with assorted meat) and 15 bites of a fruit cup; subject 8 took 29 bites of an entrée dish (pizza) and 17 bites of a pudding. In the continuous HFB activity trace, we observed an HFB increase at the time of food entering the mouth (Fig. 5A). However, an increase in the HFB activity near the time of food entering the mouth can be attributed to a number of factors, such as motor behavior of raising one’s arm, or the act of opening one’s mouth. These behaviors are likely to occur at our chosen time-lock. Thus, to evaluate food-specific responses in the insular cortex, we compared the time-locked HFB activity between eating two types of food in the same meal (Fig. 5B). Interestingly, we found significant differences in the time-locked HFB activity after individual bites of an entrée dish as compared to eating a non-entrée dish across the three subjects (*p* < 0.05, cluster-based permutation testing, *α* < 0.05). We thus categorized these sites as “food-specific”.

To assess if the changes in HFB near the time of food intake can be attributed to motor differences such as slight differences in arm positioning for eating various food, we identified electrodes positioned in the pre-motor region (Supplementary Fig. 7). No electrodes were found in the actual motor cortex in this limited cohort of subjects as depth electrode traversing the motor cortex is generally avoided. In both subjects 4 and 7, there were no pre-bite differences in HFB activity in the pre-motor region between food groups. Not surprisingly, however, we did see a dissociation in HFB activity after chewing ensued, which likely reflects food-specific motor differences during chewing. In addition, we visualized the broadband unfiltered activity (voltage) in the same contacts shown in Fig. 5B (Supplementary Fig. 8), which did not show overt signs of muscle artifacts.

**Fig. 6:**
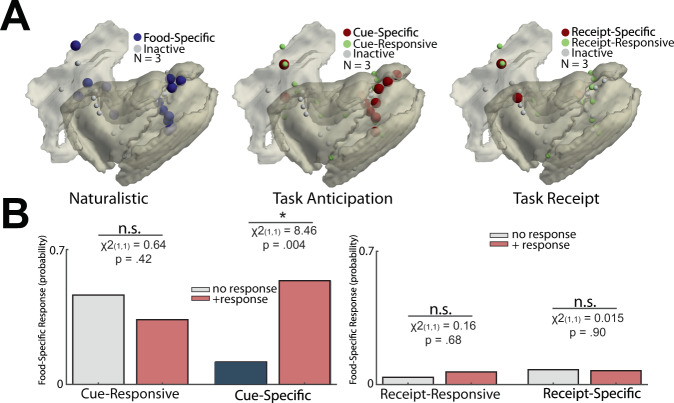
Food-specific responses during anticipation are maintained in task-based and natural eating settings. **A** Topography of response types in the insular/opercular cortex during task-based and natural eating. During eating of a standard meal, a food-specific site (blue electrodes) was defined as a significant difference in HFB time-locked activity between food types near the time that food was about to enter a subject’s mouth, whereas gray electrodes indicate a non-responsive response. During a task, a cue-specific response (red electrodes) was defined as a significant difference between palatable and neutral conditions for either anticipation or receipt of these solutions. Green electrodes (cue-responsive sites) denote significant HFB activity compared to pre-cue baseline activity, but no difference between palatable and taste-neutral conditions. Gray electrodes denote activity was not different from pre-cue baseline activity. Electrodes visualized are from the three subjects shown in Fig. 5. **B** The probability of a channel being food-specific during eating with and without significant response during task anticipation. Left: Cue-responsive contacts without discriminatory activity during anticipation were not associated with food-specific eating responses. However, the presence of cue-specific activity was significantly associated with the presence of food-specific eating responses. Right: Amongst all electrodes, neither receipt-responsive nor receipt-specific responses were associated with food-specific channels.

To understand what the food-specific sites might represent, we compared task-responsive sites with food-specific sites during a regular meal (Fig. 6). We observed the sites of food-specific eating responses and cue-specific responses were similar (Fig. 6A). Indeed, food-specific sites were significantly associated with regions demonstrating cue-specific responses (Fig. 6B, *p* = 0.004, chi-squared test, *χ*2 (1,1) = 8.46). Sites that were only cue-responsive were not associated with a food-specific eating response (Fig. 6B; *p* > 0.05, chi-squared test). In addition, receipt-responsive or receipt-specific sites were not associated with food-specific eating responses (Fig. 6B; *p* > 0.05, chi-squared test).

## Discussion

The principal objective of this study was to better understand the involvement of the insulo-opercular cortical representations in eating behavior. We used SEEG electrode recordings during a task integrating food delivery designed to elicit anticipatory and consummatory responses to food. We found evidence of food-specific encoding in the left posterior insular cortex during anticipation, but distributed and heterogeneous responses in the bilateral frontal opercular and insular cortices during receipt. Specifically, the left posterior insula showed increased activity for the taste-neutral cues. In contrast, sparse cue-specific channels in the left anterior insula showed selectivity towards the palatable cue. Single-trial classification using posterior insula HFB power dynamics during the anticipatory period yielded 64% mean TPR (and accuracy) and 69% AUC in classifying the item of anticipation. Latency analysis revealed early activation in the frontal operculum which was minimally discriminatory to food type, but this was followed by insular activation with high food-type specificity. During ad libitum meal consumption, time-locked differences in the HFB activity at the time of food was about to enter the mouth were observed between food types. In the same subjects, this response was associated with task response during anticipation, but not during receipt, providing support that the posterior insular cortex is intimately involved in food-specific expectations under both task and natural contexts.

Although the insulo-opercular cortex is generally considered the site of the primary gustatory cortex^12,17^, mounting evidence suggests the insular cortex is involved in the processing of food expectation^10,18–20^. Several studies of single-unit activity in the insular cortex of rodents have shown neurons in the area can be excited or inhibited during an auditory cue that signals food availability^10,21,22^. In addition, modulation of insular activity during a cue can subsequently alter food-oriented behavior^10^. Our results provide evidence that the human insular cortex shares similar features of cue processing. Specifically, we observed that the palatable cue could elicit both higher and lower HFB activity compared to the taste-neutral cue. Interestingly, the majority of the cue-specific responses were localized to the left posterior insula with lower HFB activity during the palatable cue. Given cue-specific HFB activity was able to correctly predict subsequent trials, there may be a translational opportunity leveraging food-specific electrographic activity to guide a neuro-modulatory approach for pathologic eating behavior, as prior preclinical studies have attempted^10,23^.

A secondary objective of our study was understanding the responses following food receipt. It is important to note that the consummatory response observed in the task paradigm is a composite sensory response, encompassing factors including taste, viscosity, and temperature. Our results are consistent with recent neuroimaging and electrophysiological studies that have found taste-responsive regions in the insular and opercular cortices^12,24,25^. In particular, 7T fMRI studies^12,25^ of taste regions in the insular cortex suggested taste encoding might be a distributed process with no insular topography specific to tastes. This is consistent with our finding that the discriminatory response during the receipt period is largely homogeneously distributed across the insulo-opercular cortex. On the contrary, our study suggests that cue-representation in the human insula is likely more localized. We found evidence that the posterior insula showed greater HFB activity for taste-neutral conditions, whereas the anterior insula showed greater HFB for palatable conditions. Interestingly, the posterior insula has been reported to respond more generally to aversive stimuli^6^ and hence the taste-neutral solution in our study may represent a relatively more aversive stimulus than the palatable solution. Similar to these studies, we found that the receipt responses were less localized and distributed across the insular and opercular regions. As milkshake has mixed macronutrients compared to the taste-neutral solution, we cannot rule out that the wide distribution of responses was due to regional encoding of other sensory aspects of the liquid presented such as viscosity and temperature.

We wished to confirm if the observed task responses were relevant to a more naturalistic setting. As neuroimaging experiments rely on a task design for repeated scans for high (signal to noise ratio) SNR and are limited by the confined scanner space, there is currently no knowledge of the activity of the human insular cortex during ad libitum eating. Taking advantage of the high temporal resolution of SEEG recordings, we found high SNR by using the time point immediately preceding food entering the mouth as a natural time-lock. Indeed, we found differential neural activity when the subject was eating an entrée as compared to non-entrée items including fruit or pudding. In addition, regions that showed discriminatory activity were significantly associated with anticipatory activity during the task, but not receipt activity. These results suggest that the insular cortex is predictably involved in the preemptive evaluation of the food we consume prior to each bite.

The laterality of insular function in food processing is debated^26^. We found evidence supporting insular laterality, as the left posterior insula has previously shown anticipatory cue-specific HFB activity changes. Similarly, several task-based fMRI studies have demonstrated asymmetric responses. One study found the left posterior insula’s response to food images was associated with serum glucose levels^27^. Another has revealed preferential left frontal operculum activation with symmetric (tip-of-tongue) application of taste stimuli^28^. In right-handed subjects, the left (dominant hemisphere) inferior insular region has been associated with taste-stimuli associated activation^29^. However, primate studies have suggested ipsilateral stimulation of nerves involved in taste sensory perception lead mainly to ipsilateral insular-opercular activation^30^. In humans, the precise connectivity of taste receptors with the insular-opercular cortices is unknown, complicated by dense innervation of the tongue gustatory papilla and other oral sensory surfaces by branches of cranial nerves V, VII, IX, and X^31^. The functional necessity of insular processing has been suggested by individual reports of patients with left posterior insular stroke^32^, leading to differential laterality in sensitivity to variations in taste intensity. These studies suggest aspects of food-related processing may be lateralized in the human insular cortex.

Prior work using gustatory and/or cue-based tasks has generated insular parcellations based on functional results. Huerta et al.^33^ aggregated the results of three food-cue paradigms, which includes the milkshake task. This study identified the left anterior insula as more consistently responsive to food cues. Similarly, a recent fMRI study incorporating food cues in addition to concurrent glucose sampling found the left dorsal insula as cue-responsive, and specifically sensitive to circulating glucose level^27^. Finally, from a structural connectivity perspective, Ghaziri et al.^34^ found that the left dorsal anterior and posterior insula is connected to the nucleus accumbens, a reward center heavily implicated in modulating the hedonic aspect of food intake. Our study adds to the literature by again demonstrating the predominance of the left insula in responding selectively to food cues. Specifically, we found the left mid to posterior insula as primarily selective, which overlaps with the ROI in many of these studies.

There is growing evidence that activity in the insular cortex is gated by physiologic needs^5^. We were not able to control for the effects of hunger and satiety on insular responses. To minimize these confounds, we conducted our task generally in the afternoon (13:00−17:00) prior to dinner. Further, while we did not measure hunger ratings, food orders were initiated and placed by the patient during standard meal times. Future naturalistic studies with built-in questionnaires to assess subjective satiety level and meal rating, as well as electromyography and eye-tracking, should be pursued to provide additional levels of behavioral control. However, as prior studies have shown saccadic eye movements mostly affect the anterior and mesial temporal lobe and are minimized with neighboring re-referencing strategies^35^, we believe the influence of ocular contamination in our study should be considered small. While we did not find an association between insular responses and individual attributes such as BMI, this may be limited by our cohort size or inadequate target sampling. In addition, the study cohort consists of individuals with medication-intractable epilepsy, and as such, cognitive processes may not generalize to the healthy population. Nevertheless, the regions of interest in our study were confirmed to be outside the seizure onset zone. In addition, we observed similar responses in individuals with different epilepsy types and severities, which supports the generalizability of our findings. Only one patient in the sample was female, which may limit generalizability due to potential sex differences. However, prior neuroimaging studies have been conducted on female samples with similar findings^5^, suggesting that these results in male patients converge with data from female samples. Lastly, electrodes were placed according to clinical indication for seizure mapping directed by safely accessible trajectories to sample insulo-opercular cortices. Thus, anatomic variations in the regions sampled exist and are unavoidable in a study of this type. According to clinical need, not all patients had bilateral or symmetric coverage of the anterior and posterior insular or associated opercular structures. To mitigate this, we performed group and individual subject analyses, which revealed consistent activity in the left posterior insula on both a group and individual level.

Here, we extended the results of a broadly used task paradigm to aspects of ad libitum eating and suggest an integral role of the insular cortex in the expectant evaluation of food. Taken together, our work provides key insight into the spatial and temporal dynamics of the human insula-opercular network during food intake.

## Methods

### Participants

Eleven human participants (two females) were implanted with at least one depth electrode in the insular/frontal opercular cortex for electrographic monitoring at Stanford University Hospital. The exact placement of electrodes (AdTech Medical) varied among participants and was determined solely based on clinical grounds following approval of a multi-disciplinary epilepsy surgery review board. Patient characteristics are described in Table 1. All patients provided individual informed consent (including the publication of the videos in journals) as approved by the Stanford University Institutional Review Board (IRB#: 11354).

### Task paradigm

Participants completed a food-reward task known as the milkshake paradigm, which is a widely used computer-based task utilized in fMRI research to assay anticipatory and consummatory responses to palatable food^11,36–40^. The task consisted of an anticipation phase and a receipt phase (Fig. 1A). During anticipation, participants saw either an image of a glass of milkshake or a glass of water that served as a visual cue signaling subsequent taste delivery. The cue lasted 1 s, followed by an appearance of a fixation cross for 2 s. To maintain participants’ attention through the task, they were also instructed to press a button when they saw the cue. At the end of 3 s, the receipt phase occurred, which involved the delivery of either 3 mL of a highly palatable solution (McDonald’s Chocolate Shake) or a water-based solution. The water-based solution consisted of 25 mM KCl and 2.5 mM NaHCO3, which was designed to mimic the natural taste of saliva^40^. Solution delivery lasted 3 s and was achieved using programmable syringe pumps (BS-8000; Braintree Scientific) attached to 60 mL syringes. Fluids traveled in sterile Tygon tubing from syringe tips to a 3D-printed mouth manifold attached to a rigid monitor arm. Participants were subsequently instructed to swallow when presented with “swallow” text. The trial order was randomized during the task, with 80 to 100 trials evenly split between palatable and taste-neutral conditions. Following completion of the task, participants were asked to rate the quality of the palatable solution (Likert scale, 1–10) and which solution (palatable vs. taste-neutral) they preferred.

### Electrode registration and cortical segmentation

Locations of electrodes in 3D space were extracted from post-implant CT, which were co-registered with patients’ pre-operative MRI^41^. Automated cortical parcellation was performed on each individual’s MRI to determine the anatomical location of the electrodes^42^. Subregions of the insular and frontal opercular cortices were divided according to the 2010 Destrieux parcellation scheme^42^: the short insular gyri, the long gyrus and the central sulcus, the anterior segment of the circular sulcus, the superior segment of the circular sulcus, the inferior segment of the circular sulcus, the pars orbitalis, the pars triangularis, and the pars opercularis (Fig. 1B). As the insular cortex has been demonstrated to have a consistent anterior-posterior division^43^, we categorized the insular cortex into anterior-posterior regions relative to the central sulcus. The anterior insular cortex included the short insular gyri, the anterior segment of the circular sulcus, and the superior segment of the circular sulcus, whereas the posterior insular cortex included the central sulcus, long gyri, and the inferior segment of the circular sulcus. The frontal opercular cortex was defined to include the inferior frontal gyrus and its three subdivisions: pars orbitalis, triangularis, and opercularis.

### Data acquisition and electrophysiological preprocessing

Stereoelectroencephalography (SEEG) recordings from implanted depth electrodes were sampled at 1024 Hz. Data preprocessing and analyses were performed using the FieldTrip toolbox^44^. Line noise (60 Hz and harmonics at 120 Hz and 180 Hz) was attenuated using a notch filter. A laplacian re-referencing scheme of flanking electrode contacts was performed as described previously to minimize far-field volume conducted contributions to the local field potential^45^. Time-frequency spectrograms were calculated using Hannings tapers. To extract the high-frequency broadband (HFB) activity, we first applied a bandpass filter (Butterworth, two-pass, 8th order). Next, we obtained the absolute value of the Hilbert transform to obtain the analytic signal, which was smoothed using boxcar averaging 200 milliseconds (ms) windows. The analytic signals of delta (1–4 Hz), theta (4–8 Hz), alpha (8–12 Hz), beta(15–25 Hz), and gamma bands (25–50 Hz) were extracted in a similar fashion to the HFB, except 4th order Butterworth filter was used as the bandpass filter. For task analyses, the data were epoched according to the onset of the cue (−2 s to 6 s, cue: 0 s, solution delivery: 3 s) and were Z-transformed against the pre-cue baseline (−0.6 to −0.1 s). For analyses of standard meals, the data were epoched according to the video-stamped time that immediately preceded food entering the mouth (−5 s to 5 s, food prior to entering the mouth: 0 s) and was Z-transformed against a pre-timelock baseline (−5 s to −4.5 s). Further, for each channel, we calculated the effect size between palatable and taste-neutral conditions for the cue period by averaging band activity between 0 s and 1 s, and for the receipt period by averaging band activity between 3 s and 4 s.

### Cue-responsive or receipt-responsive site identification

To determine if a particular electrode was responsive to cues during taste anticipation (0 s to 3 s) or delivery (3 s to 6 s) during taste receipt, we tested whether the HFB activity in the post-stimulus window was significantly larger than the baseline period (−0.6 s to −0.1 s). Differences in the post-stimulus window from baseline were tested in five consecutive, non-overlapping 500 ms windows using a cluster-based non-parametric approach^46^. This was done separately for palatable and taste-neutral conditions. A two-sample *t*-statistic was obtained at every time point and significant clusters were formed based on temporal adjacency at an alpha level of 0.05. The null distribution for the cluster *t*-statistic was produced by randomly shuffling data between the baseline window and the post-stimulus window for 1000 iterations and computing the cluster *t*-statistics. The cluster *t*-statistic was compared to this null distribution and the post-stimulus period was considered significantly different from baseline using a *p*-value of 0.05. Subsequently, a channel was considered to be either cue-responsive or receipt-responsive if a significant post-stimulus response of at least 200 ms in any time period was observed in either palatable or taste-neutral conditions.

### Cue-specific or receipt-specific site identification

After identification of responsive channels during anticipation or receipt as described above, responsive electrodes were considered either cue-specific or receipt-specific if their activity trace during anticipation (0 s to 3 s) or receipt (3 s to 6 s) was significantly different between palatable and taste-neutral conditions. To determine differences, we employed a cluster-based, non-parametric approach to compare the HFB activity between palatable and taste-neutral conditions in 500 ms time windows that previously showed a significant post-stimulus response from baseline as defined above. A two-sample t-statistic was obtained at every time point comparing palatable and taste-neutral conditions to form significant clusters based on temporal adjacency at an alpha level of 0.05. The null distribution for the cluster *t*-statistic was produced by randomly shuffling trials between the palatable and taste-neutral conditions for 1000 iterations and computing the cluster *t*-statistics. The cluster t-statistic was compared to this null distribution and the time period was considered significantly different between palatable and taste-neutral conditions based on a *p*-value of 0.05. Lastly, to prevent misclassifying potential large drifts in the signal as different in the absence of any evoked responses, a channel is considered cue-specific or receipt-specific if the time period determined to be different between the two conditions must overlap with significant post-stimulus time periods by 100 ms. To account for multiple comparisons, the *p*-threshold was adjusted via Bonferroni correction based on the number of time windows that were used for cluster-based permutation analysis (up to 5). In channels with the task-specific response, band power activity during time periods found to be significantly different on cluster-based, non-parametric testing as above, were used to calculate effect size (Cohen’s *d*).

### Classification analysis

To determine if HFB activity was sufficient to differentiate anticipation for palatable or taste-neutral taste on a single trial basis, we performed binary classification using the classification learner toolbox in MATLAB. All classifiers were initially tested using 5-fold cross-validation, default classifier parameters, and PCA to keep enough components to explain 95% of the variance. Of the tested classifiers, Kth nearest neighbor (KNN; weighted) yielded the highest performance and was fixed for all subsequent analyses. Briefly, the KNN algorithm involves measuring the distance (euclidean) between the test observation vector and all other prototypes (labeled observations) in feature space. Since the utilized model here is a weighted KNN, weights are applied to the K nearest prototypes, with lower weights applied to more distant prototypes (squared inverse distance weight). Then, the class assigned to the test observation is determined using distance-weighted voting, whereby closer prototypes contribute more to the majority vote. Default model parameters were utilized; euclidean distance metric squared inverse distance weight and 10 neighbors.

Data from posterior-insula taste-neutral selective electrodes displaying significant increases from baseline were utilized (3 subjects, 6 channels, and 250 taste-neutral and 250 palatable trials). The feature vector was defined as HFB activity in four anticipation time windows: 0–0.5, 0.5–1, 1–2, and 2–3 s. These features captured the first and second halves of the cue presentation (0–0.5, 0.5–1 s) and fixation (1-2, 2-3 s) periods, thereby approximating the transient and steady-state phases of the HFB power response dynamics for each stimulus.

KNN 5-fold cross-validation was first performed using the group data (inter-individual classification). Each fold likely contained observations from each individual’s class distributions, therefore, the test set was not composed of observations from a single patient whose data was not included in training sets (external validation). A complete external validation was not performed due to a limitation in sample size. After the generation of a group KNN model, a separate KNN model was generated for each of the three subjects (intra-individual classification).

Following the cross-validation procedure for the inter-individual classification, permutation testing was performed to assess the significance of the observed classifier performance measures including overall accuracy, and true and false-positive rates (TPR, FPR, respectively). Statistical testing was performed by generating a new model on data with shuffled values for a single feature between the two conditions. This was done 100 times, and separately for each feature. A *p*-value was obtained by examining the number of times model performance on the shuffled data was greater than (TPR) or lower than (FPR) the observed value.

### Response onset latency analysis

The time of onset for the HFB activity was determined using a technique previously described to robustly estimate response onset on a single trial level^16^. Briefly, for a given single trial, contiguous time points of the HFB activity (minimum 100 ms) above 2 standard deviations of the baseline activity were identified. At the first time point above the threshold, a 200 ms window was extracted which was further divided into 10 segments of 100 ms time series with 90% overlap. Linear regression was performed on each of the 20 segments to obtain slope and residual error. Segments with the top five slopes were selected, and the segment with the least mean squared error was defined as the “onset” segment. The first time point of onset segment was used to define the response onset latency (ROL) for the single trial. This procedure was carried out separately for anticipation and receipt periods. For each region of interest, we computed the ROL for all trials, irrespective of palatable or taste-neutral condition, and for all channels that fell within the anatomical region. Hence, the cumulative number of ROL per region is the product of the individual trial and channel numbers.

### ad libitum consumption analysis

We hypothesized that regions demonstrating cue-specific responses might exhibit similar behavior under a naturalistic setting. Hence, we selected subjects with coverage in regions showing cue-specific responses to examine simultaneous video (29.97 Hz framerate) and SEEG recordings as they ate their daily meals. In order to obtain high signal-to-noise ratio (SNR) responses outside of a task paradigm, we looked for a consistent behavior during ad libitum consumption for electrophysiologic time-locking. During the process of eating, a universal movement is to bring the food up to the mouth for consumption. Hence, we defined that the time point immediately preceding food entry (just as the food is about to enter the mouth) as a visual time-lock for our offline analyses. To test this, we chose a meal video segment for analysis using the following inclusion criteria: (1) the subject’s face must be visible in the video to allow for tracking of food movement; (2) the subject must be eating a meal which consists of at least two types of food (e.g., entrée or dessert) for comparison, such that each one may serve as a control for common motor signals that may be time-locked; (3) there must be at least 10 repetitions in each food type to allow for trial-averaging; (4) there must be no seizures or epileptic activity both 5 h before and after the video file to avoid preictal or postictal activity; (5) when multiple videos are eligible, we chose the video meal segment that was closest in time to when the task recording was performed to avoid mismatch in signal quality between task and naturalistic conditions^47^. Two independent reviewers (AF, RS) evaluated the eating content of the video segment by time-stamping the immediate timepoint prior to every food bite. Finally, as meal segments can be potentially long in duration and susceptible to inter-ictal activity, we applied a spike detection and interpolation process. Using FieldTrip toolbox’s artifact rejection module, large inter-ictal spikes were identified by applying a standard deviation threshold to the 20–50 Hz^48^ bandpass filtered signal averaged across channels. The 100 ms signal surrounding the identified spike was replaced with stationary SEEG time series that represented an average amplitude and spectral profile as the background signal^49^.

### Identification of food-specific sites during natural eating

To determine if there was a significant food-specific response in the insular/frontal opercular cortices during regular meals, we compared the time-locked HFB activity between two food types near the time preceding food entry into the mouth. Due to the quality of the recorded videos, the only entrée vs. non-entrée food items were clearly discernable. Non-entrée food items included either pudding or fruit. To compare the HFB activity between two food types, we used cluster-based non-parametric testing to obtain two-sample t-statistic at every time point from −1 s to 1 s between entrée or non-entrée trials. Significant clusters were formed based on temporal adjacency at an alpha level of 0.05. The null distribution for the cluster *t*-statistic was produced by randomly shuffling trials between the entrée and non-entrée trials for 1000 iterations and computing the cluster *t*-statistics. The cluster *t*-statistic was compared to this null distribution and the time period was considered significantly different between the two conditions based on a *p*-value of 0.05. Food type-specific HFB activity response during meal consumption was investigated in an effort to control for potential stereotypic responses representative of motor system activation leading up to food bites. Finally, to understand what the difference in the HFB activity between eating two food types may represent, we performed a chi-square analysis to test for associations in the responses between task-based and ad libitum consumption.

### Reporting summary

Further information on research design is available in the Nature Research Reporting Summary linked to this article.

## Supplementary information

Supplementary Information

Reporting Summary

## Data Availability

The data that support the findings of this study are available from the corresponding author on reasonable request. Source data are provided with this paper.

## Source data

Source Data

## Code availability

Matlab 2019b was used to perform all electrophysiologic analyses in this study. The dependent toolboxes used were: Fieldtrip-20190828, SPM8 (2013-09-17), SPM12 (2018-11-07), iELVis (2019-09-29), Matlab Signal Processing toolbox. No new algorithm or pre-processing techniques were performed outside of standard toolbox usages. The code for analyzing the data of this study is available from the corresponding author on reasonable request.
