# Supplementary material for: The insulo-opercular cortex encodes food-specific content under controlled and naturalistic conditions
Source: Nat Commun. 2021 Jun 14;12:3609. doi: 10.1038/s41467-021-23885-4 (PMC8203663; doi:10.1038/s41467-021-23885-4)
Supplement: Supplementary file 1 — Supplementary Information [file 41467_2021_23885_MOESM1_ESM.pdf]

Supplementary Information

The Insulo-Opercular Cortex Encodes Food-specific Content under Controlled and Naturalistic Conditions

Huang et al.,

**Supplementary Table 1: Correlation between task responses and subject attributes**			
**Type of Correlation**	**N (subjects)**	**R**	**P**
Cue-Responses			
% Responsive – BMI	11	-0.06	0.87
% Specific – BMI	11	-0.38	0.24
% Responsive – MS rating	11	0.46	0.16
% Specific – MS rating	11	0.58	0.06
% Responsive – Age	11	0.21	0.54
% Specific – Age	11	0.10	0.77
Receipt- Responses			
% Responsive – BMI	11	0.06	0.86
% Specific – BMI	11	-0.37	0.29
% Responsive – MS rating	11	0.18	0.60
% Specific – MS rating	11	-0.03	0.93
% Responsive – Age	11	0.09	0.79
% Specific – Age	11	-0.28	0.44

Supplementary Table 2: MNI coordinates of cue and/or receipt-specific responses					
**#**	**Contact Location**	**Response**	**x**	**y**	**z**
1	L Anterior Circular Sulcus	Cue-Specific<br>Receipt-Specific	-27.72	17.69	-3.39
2	R Superior Circular Sulcus	Cue-Specific	33.67	15.36	13.84
3	L Short Insular Gyri	Cue-Specific	-40.19	14.94	-5.21
4	L Frontal Operculum	Cue-Specific<br>Receipt-Specific	-44.86	26.26	19.93
5	L Long Gyrus and Central Sulcus	Cue-Specific	-37.87	-6.78	1.83
6	L Long Gyrus and Central Sulcus	Cue-Specific	-37.02	-5.16	4.87
7	L Long Gyrus and Central Sulcus	Cue-Specific<br>Receipt-Specific	-36.16	-3.54	7.92
8	L Short Insular Gyri	Cue-Specific	-34.28	13.10	-4.16
9	L Superior Circular Sulcus	Cue-Specific	-27.02	21.73	9.12
10	L Long Gyrus and Central Sulcus	Cue-Specific	-38.93	-10.27	10.54
11	L Long Gyrus and Central Sulcus	Cue-Specific	-39.07	-13.73	13.22
12	L Long Gyrus and Central Sulcus	Cue-Specific	-39.20	-17.19	15.91
13	L Frontal Operculum	Cue-Specific	-32.97	25.96	7.28
14	L Inferior Circular Sulcus	Cue-Specific	-42.12	-8.95	-5.99
15	L Inferior Circular Sulcus	Cue-Specific	-41.01	-9.09	-0.50
16	L Long Gyrus and Central Sulcus	Cue-Specific	-40.07	-9.16	5.086
17	L Anterior Circular Sulcus	Cue-Specific	-30.59	15.90	12.35
18	L Short Insular Gyri	Cue-Specific	-36.47	9.16	-11.71
19	L Short Insular Gyri	Cue-Specific	-35.83	10.91	-9.51
20	R Long Gyrus and Central Sulcus	Receipt-Specific	40.60	3.52	-8.37
21	L Superior Circular Sulcus	Receipt-Specific	-31.68	20.54	13.72
22	L Frontal Operculum	Receipt-Specific	-38.26	20.02	6.57
23	L Superior Circular Sulcus	Receipt-Specific	-28.81	19.51	5.81
24	L Long Gyrus and Central Sulcus	Receipt-Specific	-36.38	-8.65	6.45
25	R Superior Circular Sulcus	Receipt-Specific	36.55	13.86	-4.22
26	R Superior Circular Sulcus	Receipt-Specific	35.26	14.58	-0.16
27	R Short Insular Gyri	Receipt-Specific	41.69	4.69	2.59
28	R Superior Circular Sulcus	Receipt-Specific	34.51	-8.13	10.80
29	R Inferior Circular Sulcus	Receipt-Specific	38.01	-21.08	4.72

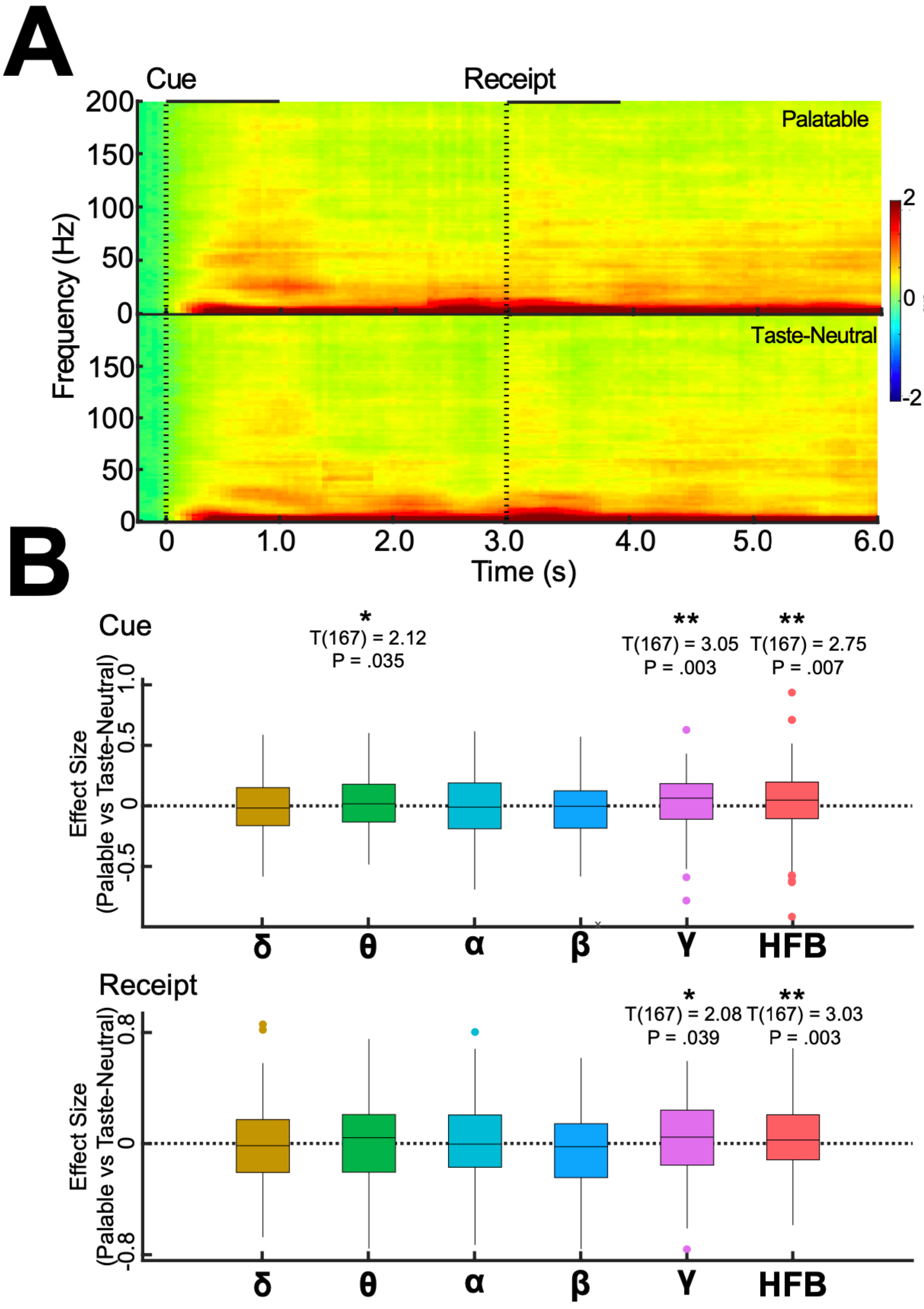

A
Cue
Receipt
Palatable
Taste-Neutral
Frequency (Hz)
200
150
100
50
0
db
2
0
-2
Time (s)
0
1.0
2.0
3.0
4.0
5.0
6.0
B
Cue
*
T(167) = 2.12
P = .035
**
T(167) = 3.05
P = .003
**
T(167) = 2.75
P = .007
Effect Size
(Palable vs Taste-Neutral)
1.0
0.5
0
-0.5
δ
θ
α
β
γ
HFB
Receipt
*
T(167) = 2.08
P = .039
**
T(167) = 3.03
P = .003
0.8
0
-0.8

Supplementary Figure 1: Time-frequency spectrogram for mean insulo-opercular activity and effect size quantification by conventional frequency ranges. A) Time-frequency spectrogram computed separately for palatable and taste-neutral conditions using all insulo-opercular channels (N = 168 channels). The vertical dashed line denotes the onset of the solution cue (t=0s) and solution delivery (t=3s). **B)** Boxplots showing effect size (Cohen's d) calculated between palatable and taste-neutral conditions across all insulo-opercular channels (N = 168) in each conventional frequency bands: delta (1-4Hz), theta (4-8Hz), alpha (8-12Hz), beta(15-25Hz), gamma bands (25-50Hz) and HFB (70-170Hz). The first second after stimulus onset was used. Significantly higher effect size (favoring palatable conditions) was found in gamma and HFB power after cue presentation and solution delivery. Theta power during anticipation was also associated with significantly higher effect size favoring the palatable condition. One-sample T-test was employed for each band, without correction for multiple comparison. For each boxplot, the center defines 50% of the data, whereas the upper and lower box defines 25% and 75% of the data respectively. The end of the whiskers corresponds to +/- 2.7 standard deviation.

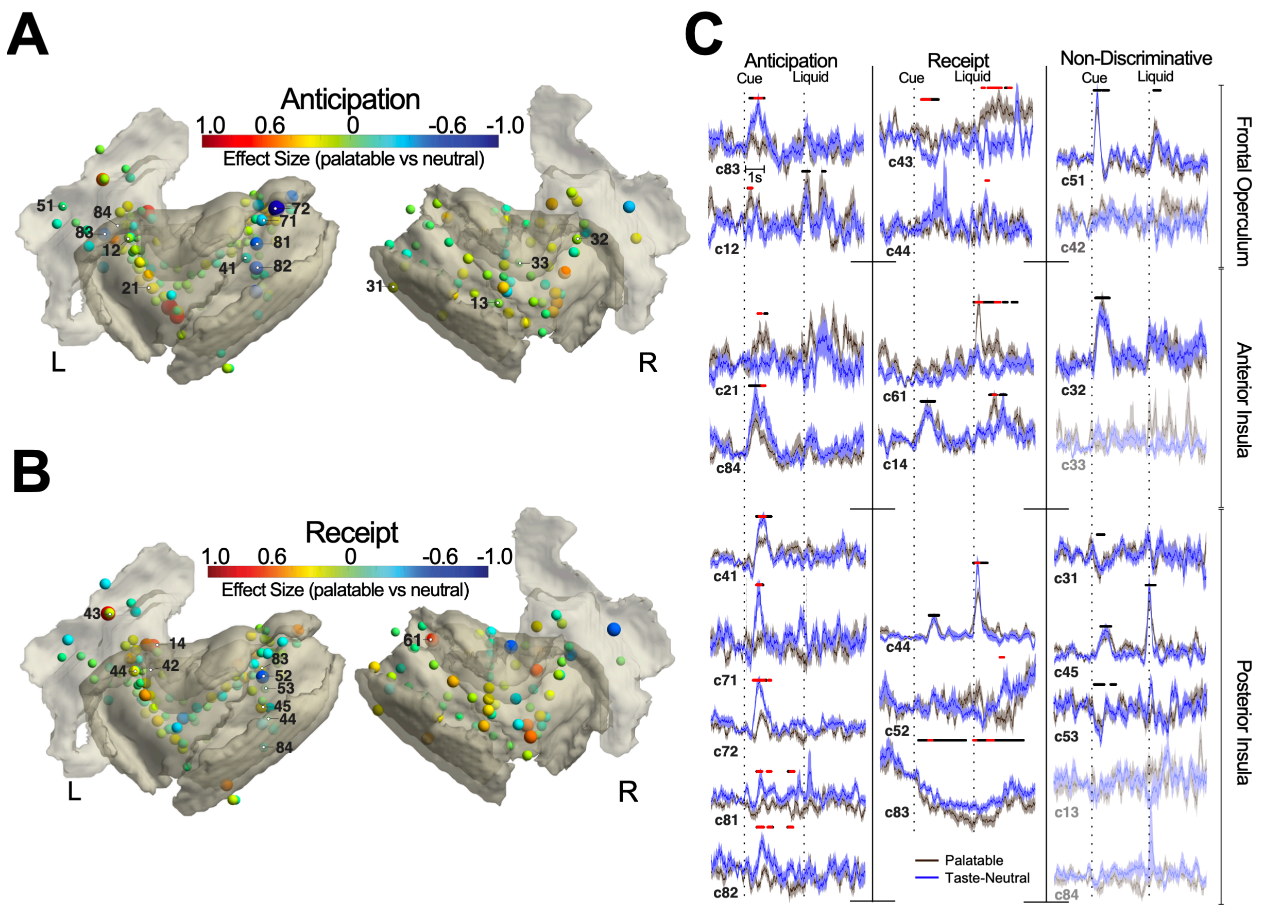

Supplementary Figure 2: Site-by-site differential insulo-opercular activity when anticipating and receiving task solutions. A) Unthresholded site-by-site differences in anticipatory neural activity as measured by effect size (Cohen's D) calculated using average HFB within 1s of cue onset. Shades of red indicate greater response in palatable trials whereas shades of blue indicate greater response in taste-neutral trials. **B)** Same set of plots as in **(A)** showing site-by-site differences in receipt neural activity. **C)** Mean HFB waveforms in exemplar sites that show either discriminative neural activity during anticipation and receipt, or non-discriminatory activity. Time points significantly different between palatable and neutral conditions ($p<0.05$, cluster-based permutation testing, alpha < 0.05) are marked in red along the horizontal axis, while time points significantly above the pre-cue baseline activity are marked in black ($p<0.05$, cluster-based permutation testing, alpha < 0.05). Transparent lines indicate activity was not different from pre-cue baseline activity. The vertical dashed line denotes the onset of the solution cue (t=0) and solution delivery (t=3). Error bars show ±SEM.

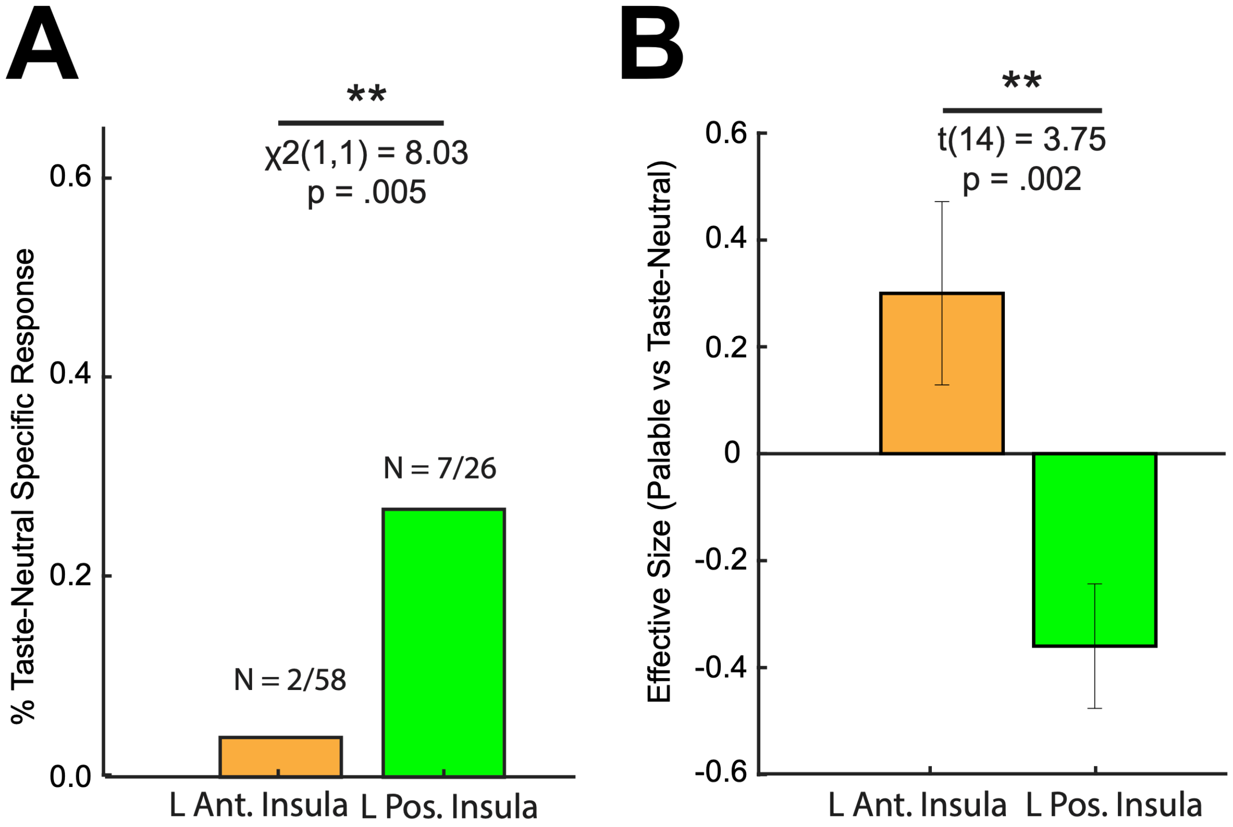

Supplementary Figure 3: Proportion taste-neutral specific channels and effect size in the left anterior and posterior insula. A) The proportion of cue-specific channels that are favored toward the taste-neutral cue was significantly higher in the left posterior insula (N = 7/26, 27%) as compared to the left anterior insula (N = 2/58, 3%). Chi-square proportion test was employed. **B)** Mean effect size of cue-specific channels in the left anterior insula as compared to the left posterior insula. The left anterior insula had a mean positive effect size (N = 7 cue-specific channels), thus favoring the palatable cue. This is significantly different than the mean negative effect size found in the left posterior insula (N = 9 cue-specific channels). Two-sample T-test was employed. Error bars show ±SEM.

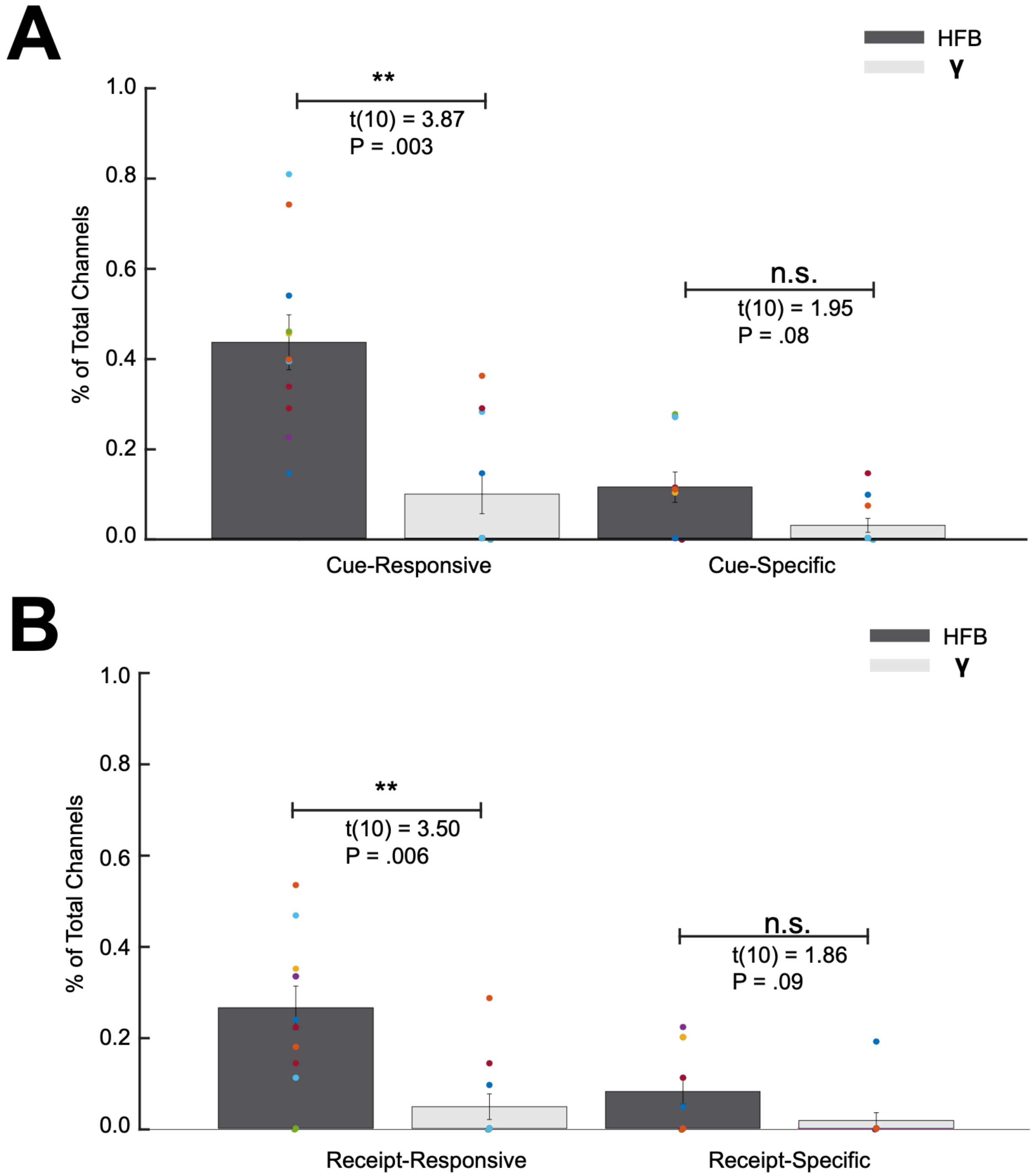

Supplementary Figure 4: Proportion of task-responsive and task-specific electrodes as determined by HFB (70-170 Hz) or gamma (25-50 Hz) activity. Each colored dot represents a single subject (N = 11 subjects). **A)** Proportion of cue-responsive and cue-specific channels out of total channels as indexed by HFB or gamma power. A significantly higher proportion of cue-responsive channels was found using HFB as compared to gamma activity. The proportion of cue-specific channels determined by HFB was also higher than that determined by gamma

activity, although this was not significant**. B)** Proportion of receipt-responsive and receipt-specific channels out of total channels as indexed by HFB or gamma power. A significantly higher proportion of receipt-responsive channels was found using HFB as compared to gamma activity. The proportion of receipt-specific channels determined by HFB was also higher than that determined by gamma activity, although not significant. Paired T-test was employed. HFB: high-frequency broadband. Error bars show ±SEM.

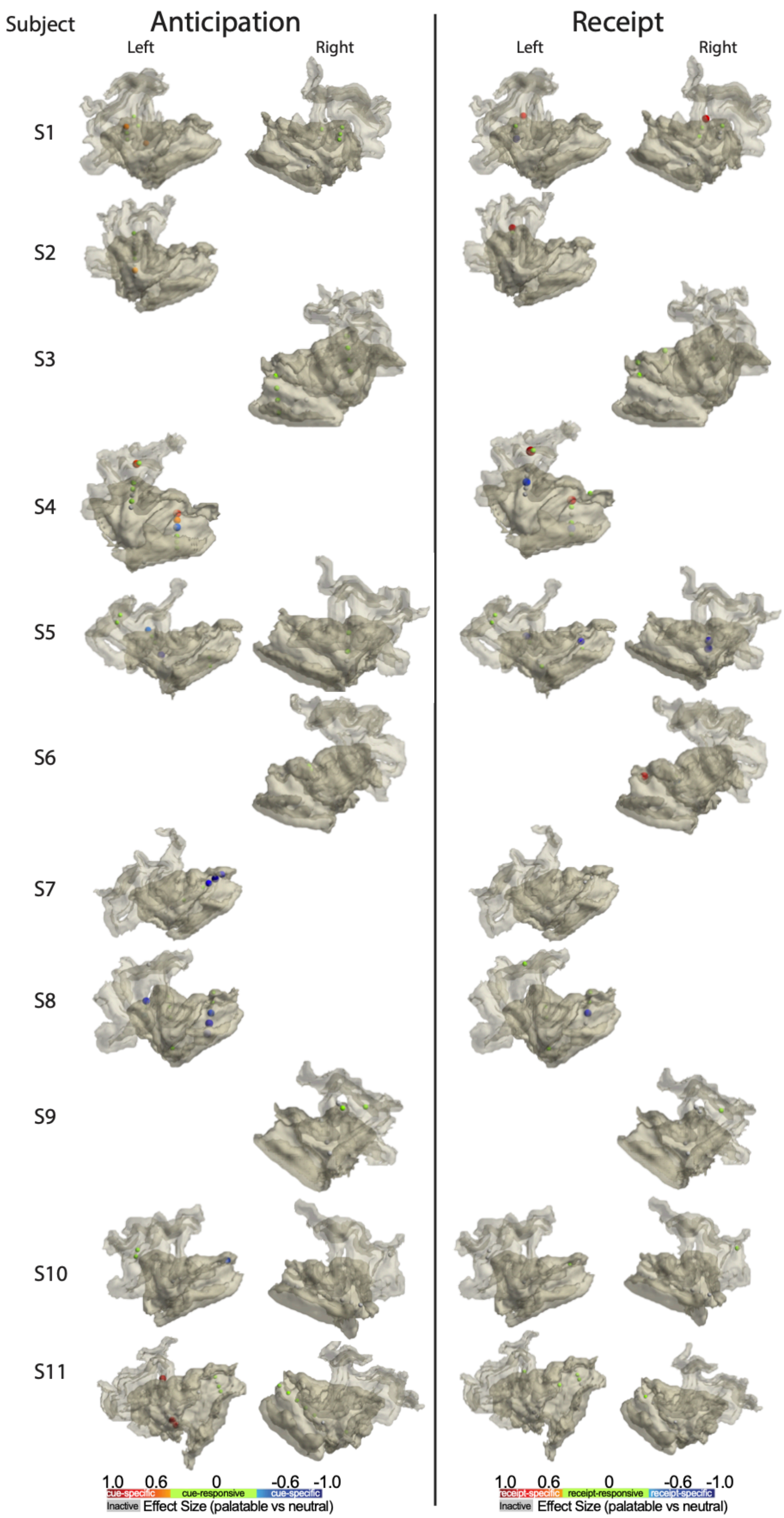

Subject
Anticipation
Receipt
Left
Right
Left
Right
S1
S2
S3
S4
S5
S6
S7
S8
S9
S10
S11
1.0 0.6 0 -0.6 -1.0
cue-specific
cue-responsive
cue-specific
Inactive
Effect Size (palatable vs neutral)
1.0 0.6 0 -0.6 -1.0
receipt-specific
receipt-responsive
receipt-specific
Inactive
Effect Size (palatable vs neutral)

Supplementary Figure 5: Individual subject parcellation of insular and frontal opercular cortices and responses during the milkshake paradigm. Automatic parcellation of the insular cortex was performed using Freesurfer. Electrodes responses were categorized as inactive (grey), cue or receipt-responsive (green), or cue or receipt-specific (shades of red or blue respectively).

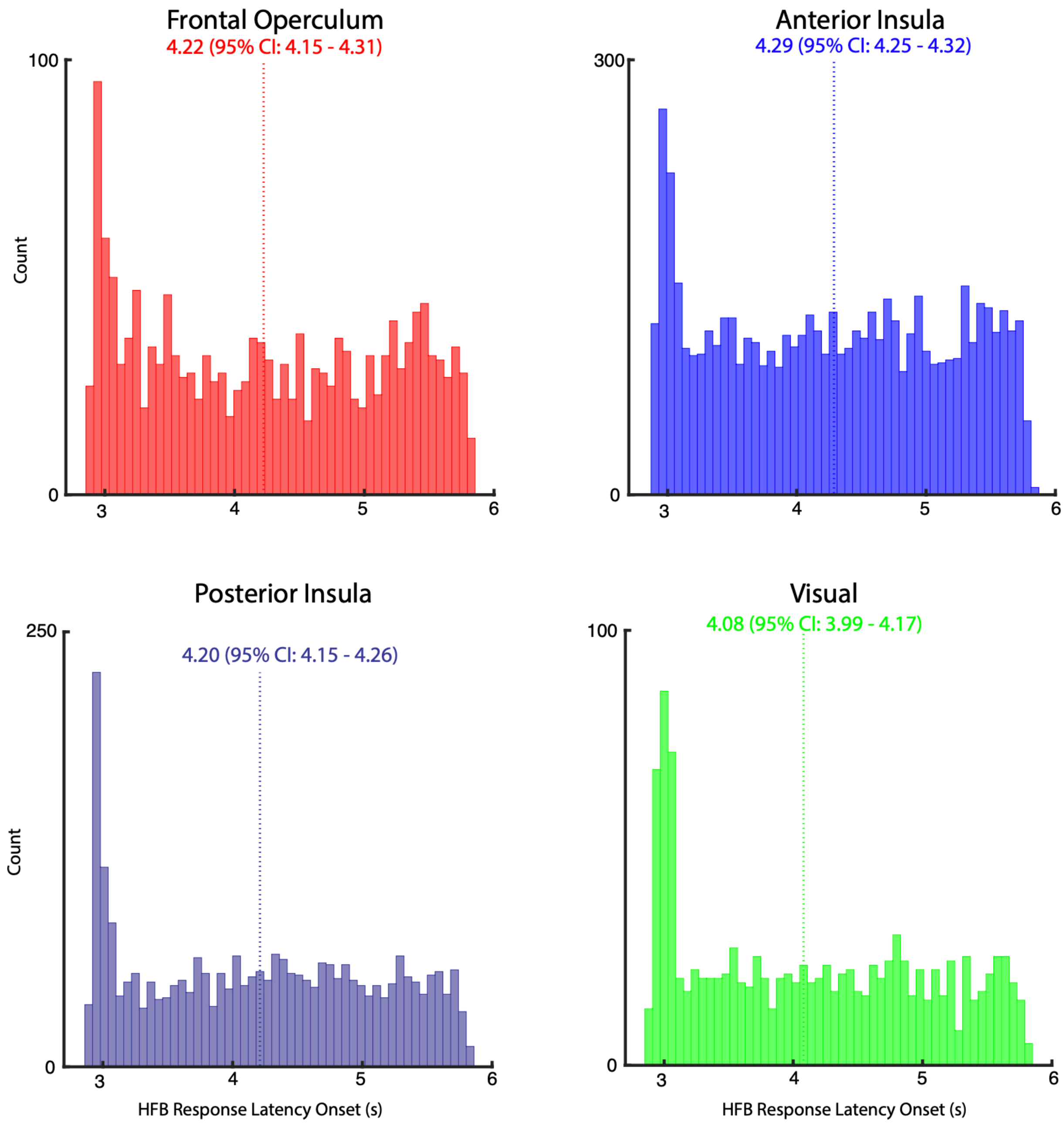

Supplementary Figure 6: Comparison of HFB response onset by anatomical location during task receipt. Histogram showing distribution of ROL of HFB at the visual region, the frontal operculum, the anterior insula, the posterior insula, and visual regions. Each observation represents the ROL of HFB at the single trial level calculated for all trials across all subjects (palatable and neutral conditions). The vertical line denotes the median ROL time.

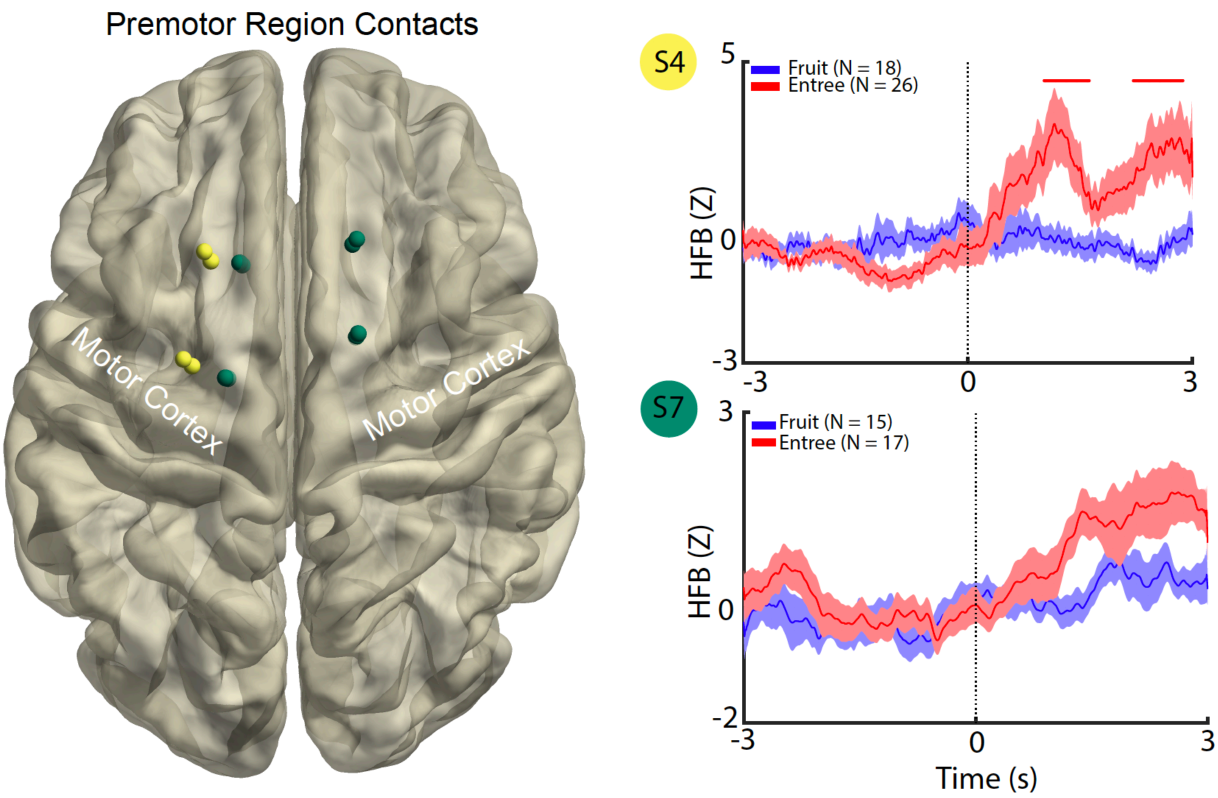

Supplementary Figure 7: Time-locked HFB activity in the pre-motor region during natural eating stratified by the pre-bite and post-bite (i.e. chewing) periods. Two subjects had superficial electrodes in the pre-motor region (S4, yellow contacts, N = 4; S7, green contacts, N = 8). For each subject, the mean time-locked waveforms stratified by type of food consumed are shown. Dotted vertical lines denote the immediate time point as food was about to enter the subject's mouth. Time points significantly different between entrée versus fruit near the time of food entering the mouth ($p < 0.05$, cluster-based permutation testing, alpha < 0.05) are marked in red along the horizontal axis. Error bars show ±SEM.

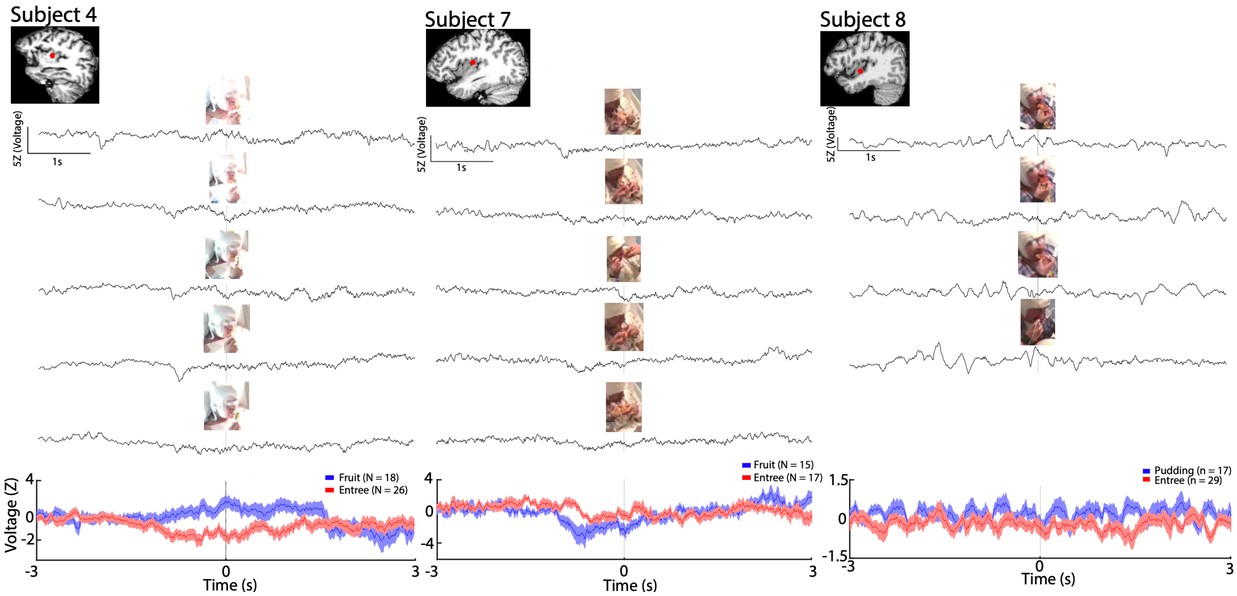

Supplementary Figure 8: Single trial broadband (voltage) activity and time-locked voltage activity during natural eating stratified by type of food consumed. Single trial and mean voltage activity from spike-minimized data are shown from the same exemplar contacts in Figure 5 (left posterior insula). The same events visualized in Figure 5 were shown here magnified to include -3s and +3s from the time of food intake. Dotted vertical lines denote the immediate time point as food was about to enter the subject's mouth (time 0).
