# Supplementary material for: The insulo-opercular cortex encodes food-specific content under controlled and naturalistic conditions
Source: Nat Commun. 2021 Jun 14;12:3609. doi: 10.1038/s41467-021-23885-4 (PMC8203663; doi:10.1038/s41467-021-23885-4)
Supplement: Supplementary file 2 — Reporting Summary [file 41467_2021_23885_MOESM2_ESM.pdf]

nature research

Corresponding author(s): Casey Halpern

Last updated by author(s): 04/10/2021

Reporting Summary

Nature Research wishes to improve the reproducibility of the work that we publish. This form provides structure for consistency and transparency in reporting. For further information on Nature Research policies, see our Editorial Policies and the Editorial Policy Checklist.

Statistics

For all statistical analyses, confirm that the following items are present in the figure legend, table legend, main text, or Methods section.

n/a	Confirmed	
☐	☒	The exact sample size (n) for each experimental group/condition, given as a discrete number and unit of measurement
☐	☒	A statement on whether measurements were taken from distinct samples or whether the same sample was measured repeatedly
☐	☒	The statistical test(s) used AND whether they are one- or two-sided<br>*Only common tests should be described solely by name; describe more complex techniques in the Methods section.*
☐	☒	A description of all covariates tested
☐	☒	A description of any assumptions or corrections, such as tests of normality and adjustment for multiple comparisons
☐	☒	A full description of the statistical parameters including central tendency (e.g. means) or other basic estimates (e.g. regression coefficient) AND variation (e.g. standard deviation) or associated estimates of uncertainty (e.g. confidence intervals)
☐	☒	For null hypothesis testing, the test statistic (e.g. F, t, r) with confidence intervals, effect sizes, degrees of freedom and P value noted<br>*Give P values as exact values whenever suitable.*
☒	☐	For Bayesian analysis, information on the choice of priors and Markov chain Monte Carlo settings
☐	☒	For hierarchical and complex designs, identification of the appropriate level for tests and full reporting of outcomes
☐	☒	Estimates of effect sizes (e.g. Cohen's d, Pearson's r), indicating how they were calculated

Our web collection on statistics for biologists contains articles on many of the points above.

Software and code

Policy information about availability of computer code

Data collection	Electrophysiologic data was obtained from intracranially placed depth electrodes (AdTech Medical) by using the clinically available Nihon Kohden EEG recording system.
Data analysis	Matlab 2019b was used to perform all electrophysiologic analyses in this study. The dependent toolboxes used were: Fieldtrip-20190828, SPM8 (2013-09-17), SPM12 (2018-11-07), iELVis (2019-09-29), Matlab Signal Processing toolbox. No new algorithm or pre-processing techniques were performed outside of standard toolbox usages. The code for analyzing the data of this study are available from the corresponding author on reasonable request.

For manuscripts utilizing custom algorithms or software that are central to the research but not yet described in published literature, software must be made available to editors and reviewers. We strongly encourage code deposition in a community repository (e.g. GitHub). See the Nature Research guidelines for submitting code & software for further information.

Data

Policy information about availability of data

All manuscripts must include a data availability statement. This statement should provide the following information, where applicable:

- Accession codes, unique identifiers, or web links for publicly available datasets
- A list of figures that have associated raw data
- A description of any restrictions on data availability

The data that support the findings of this study are available from the corresponding author on reasonable request.

Field-specific reporting

Please select the one below that is the best fit for your research. If you are not sure, read the appropriate sections before making your selection.

- [ ] Life sciences
- [x] Behavioural & social sciences
- [ ] Ecological, evolutionary & environmental sciences

For a reference copy of the document with all sections, see nature.com/documents/nr-reporting-summary-flat.pdf

Behavioural & social sciences study design

All studies must disclose on these points even when the disclosure is negative.

Study description	This was a quantitative prospective study where we enrolled 11 consecutive patients with intracranial depth electrodes targeting the insulo-opercular cortex placed for sole purposes of clinical monitoring. These patients were asked to participate in a cognitive task involving viewing a food cue and subsequently receiving a taste of either taste-neutral or palatable solution.
Research sample	11 consecutive human participants (2 female) with coverage of the insulo-opercular cortex were enrolled. The baseline demographics are included in Table 1. Briefly, Age ranged from 20 to 62, BMI ranged 19 to 51 with no seizure onset zone within the insulo-opercular region.
Sampling strategy	Consecutive enrollment of patients meeting the enrollment criteria (intracranial electrode targeting the insulo-opercular cortex). The sample size was chosen to allow for adequate coverage of the insulo-opercular cortex, as well as based on clinical restraints. We have included all possible subjects meeting inclusion criteria since the inception of the study.
Data collection	Nihon Kohden EEG-1200 and its supplied software was used for all video and EEG data capture in this study. No blinding was performed in this study. The researchers instructed the participants on the tasks and operated the electrophysiology hardware and software.
Timing	The subject enrollment period was from 10/2018 to 10/2020
Data exclusions	Subjects with lesions in the brain including mass, infarct and encephalomalacia were not enrolled in the study
Non-participation	No enrolled subjects declined to participant.
Randomization	No randomization process apply to this study as there was no intervention. All subjects participated in the study task.

Reporting for specific materials, systems and methods

We require information from authors about some types of materials, experimental systems and methods used in many studies. Here, indicate whether each material, system or method listed is relevant to your study. If you are not sure if a list item applies to your research, read the appropriate section before selecting a response.

Materials & experimental systems

n/a	Involved in the study	
[x]	[ ]	Antibodies
[x]	[ ]	Eukaryotic cell lines
[x]	[ ]	Palaeontology and archaeology
[x]	[ ]	Animals and other organisms
[ ]	[x]	Human research participants
[x]	[ ]	Clinical data
[ ]	[x]	Dual use research of concern

Methods

n/a	Involved in the study	
[x]	[ ]	ChIP-seq
[x]	[ ]	Flow cytometry
[x]	[ ]	MRI-based neuroimaging

Human research participants

Policy information about studies involving human research participants

Population characteristics	We recorded the age, sex, handedness, BMI, rating of the palatable solution as well as seizure onset zone for each participant. Prior fMRI literature has suggested BMI may influence brain activity to palatable vs taste-neutral cue. Further, avoiding signal within the seizure onset zone can limit inter-ictal activity contamination.
Recruitment	Consecutive subjects meeting inclusion criteria were asked if they were interested in participating in this study during their hospitalization stay for intracranial seizure monitoring. No patients declined to participate which limits any self-selection bias. Subjects who has known lesions in their brain (encephalomalacia, mass, acute infarct) were excluded from enrollment as results from these subjects may be difficult to generalize to the broader population.
Ethics oversight	Stanford University IRB

Note that full information on the approval of the study protocol must also be provided in the manuscript.

Dual use research of concern

Policy information about dual use research of concern

Hazards

Could the accidental, deliberate or reckless misuse of agents or technologies generated in the work, or the application of information presented in the manuscript, pose a threat to:

No	Yes	
☒	☐	Public health
☒	☐	National security
☒	☐	Crops and/or livestock
☒	☐	Ecosystems
☒	☐	Any other significant area

Experiments of concern

Does the work involve any of these experiments of concern:

No	Yes	
☒	☐	Demonstrate how to render a vaccine ineffective
☒	☐	Confer resistance to therapeutically useful antibiotics or antiviral agents
☒	☐	Enhance the virulence of a pathogen or render a nonpathogen virulent
☒	☐	Increase transmissibility of a pathogen
☒	☐	Alter the host range of a pathogen
☒	☐	Enable evasion of diagnostic/detection modalities
☒	☐	Enable the weaponization of a biological agent or toxin
☒	☐	Any other potentially harmful combination of experiments and agents
